# Early treated HIV-infected children remain at risk of growth retardation during the first five years of life: Results from the ANRS-PEDIACAM cohort in Cameroon

**DOI:** 10.1371/journal.pone.0219960

**Published:** 2019-07-18

**Authors:** Casimir Ledoux Sofeu, Mathurin Cyrille Tejiokem, Calixte Ida Penda, Camelia Protopopescu, Francis Ateba Ndongo, Suzie Tetang Ndiang, Georgette Guemkam, Josiane Warszawski, Albert Faye, Roch Giorgi, the ANRS-PEDIACAM study group

**Affiliations:** 1 Centre Pasteur du Cameroun, Service d’épidémiologie et de santé publique, Yaoundé, Cameroun; 2 Aix-Marseille Univiversité, INSERM, IRD, SESSTIM, Sciences Economiques & Sociales de la Santé & Traitement de l’Information Médicale, France; 3 Université de Bordeaux, ISPED, INSERM Bordeaux Population health U1219 (Biostatistic), France; 4 Université de Douala, Faculté de Médecine et de Sciences Pharmaceutiques, Cameroun; 5 Hôpital de Jour, Hôpital Laquintinie, Douala, Cameroun; 6 Centre Mère et Enfant de la Fondation Chantal Biya, Yaoundé, Cameroun; 7 Centre Hospitalier d’Essos, Yaoundé, Cameroun; 8 INSERM U1018 (CESP)—Equipe 4 (VIH et IST), Le Kremlin Bicêtre, France; 9 Assistance Publique des Hôpitaux de Paris, Service d’Epidémiologie et de Santé Publique, Hôpital de Bicêtre, Le Kremlin Bicêtre, France; 10 Université de Paris Sud 11, Paris, France; 11 Assistance Publique des Hôpitaux de Paris, Pédiatrie Générale, Hôpital Robert Debré, Paris, France; 12 Université Paris 7 Denis Diderot, Paris Sorbonne Cité, Paris, France; 13 INSERM UMR 1123 (ECEVE), France; 14 APHM, Hôpital de la Timone, Service Biostatistique et Technologies de l’Information et de la Communication, Marseille, France; The Ohio State University, UNITED STATES

## Abstract

**Background:**

Long-term growth in HIV-infected infants treated early in resource-limited settings is poorly documented. Incidence of growth retardation, instantaneous risk of death related to malnutrition and growth parameters evolution during the first five years of life of uninfected and early treated HIV-infected children were compared and associated factors with growth retardation were identified.

**Methods:**

Weight-for-age (WAZ), weight-for-length (WLZ), and length-for-age (LAZ) Z-scores were calculated. The ANRS-PEDIACAM cohort includes four groups of infants with three enrolled during the first week of life: HIV-infected (HI, n = 69), HIV-exposed uninfected (HEU, n = 205) and HIV-unexposed uninfected (HUU, n = 196). The last group included HIV-infected infants diagnosed before 7 months of age (HIL, n = 141). The multi-state Markov model was used to describe the incidence of growth retardation and identified associated factors.

**Results:**

During the first 5 years, 27.5% of children experienced underweight (WAZ<-2), 60.4% stunting (LAZ<-2) and 41.1% wasting (WLZ<-2) at least once. The instantaneous risk of death observed from underweight state (35.3 [14.1–88.2], 84.0 [25.5–276.3], and 6.0 [1.5–24.1] per 1000 person-months for 0–6 months, 6–12 months, and 12–60 months respectively) was higher than from non-underweight state (9.6 [5.7–16.1], 20.1 [10.3–39.4] and 0.3 [0.1–0.9] per 1000 person-months). Compared to HEU, HIL and HI children were most at risk of wasting (adjusted HR (aHR) = 4.3 (95%CI: 1.9–9.8), P<0.001 and aHR = 3.3 (95%CI: 1.4–7.9), P = 0.01 respectively) and stunting for HIL (aHR = 8.4 (95%CI: 2.4–29.7). The risk of underweight was higher in HEU compared to HUU children (aHR = 5.0 (CI: 1.4–10.0), P = 0.001). Others associated factors to growth retardation were chronic pathologies, small size at birth, diarrhea and CD4< 25%.

**Conclusions:**

HIV-infected children remained at high risk of wasting and stunting within the first 5 years period of follow-up. There is a need of identifying suitable nutritional support and best ways to integrate it with cART in pediatric HIV infection global care.

## Introduction

Growth retardation is a major public health problem, mainly in children aged less than five year of age, with an estimated 3.1 million deaths annually, accounting for 45% of all child deaths [1, 2]. In the last few years, about half of global under-five deaths occurred in sub-Saharan Africa (SSA), with more than 50% attributable to malnutrition [3, 4]. Moreover, SSA is also impacted by HIV infection as more than 90% of the 1.8 million children (< 15 years) living with HIV globally currently reside at the end of 2017, with only 52% of them on antiretroviral therapy (ART). During that period, there were 110,000 AIDS-related deaths among children below the age of 15 [5, 6]. In Cameroon, the estimated number of new pediatric HIV infections was 4500 in 2017 and vertical transmission rate at 13% after breastfeeding ends[5, 7]. Prevalence of stunting, underweight, and wasting among children under 5 years in 2014 was 31.7%, 14.8%, and 5.2% respectively [8].

Malnutrition is a common comorbidity among HIV-infected children and HIV infection is also diagnosed frequently in children with severe malnutrition [9]. These two pathologic conditions interact and can each impede physical growth, cognitive, and social development of the child [4, 10]. ART initiation can help to resolve malnutrition as shown by many authors [11–14]. But, growth improvement seems to be greater for children initiated early on ART compared with those initiated at older ages [11–13]. Also, some authors indicated that nutritional status of a certain number of HIV-infected children on ART will not be completely restored even with significant gain on weight and/or height [12, 15, 16]. Long-term growth in HIV-infected infants initiated early on cART in resource-limited settings is poorly documented [13]. Following this observation and having an ongoing cohort constituted of early treated HIV-infected and HIV-uninfected children with longer follow-up periods, we found it necessary to conduct this study assuming a long-term direct benefit of early ART on growth catch-up.

We compared incidence of growth retardation, instantaneous risk of death related to growth retardation, and growth evolution over the first 5 years of life of early treated HIV-infected and HIV-uninfected children born to HIV positive or negative mothers and identified factors associated with growth retardation in Cameroon.

## Methods

### Ethics statement

The ANRS-PEDIACAM study was granted ethical approval in Cameroon by the National Ethics Committee and in France by the Biomedical Research Committee of the Pasteur Institute of Paris. The Cameroon Ministry of Public Health gave administrative authorization to start the study. Written informed consent was obtained from parents or guardians prior to inclusion of infants into the research project

### Data source

Data used in this analysis were obtained from the ANRS-PEDIACAM, an ongoing prospective observational study based in three referral hospitals in Cameroon (The Maternity of the Central hospital/Mother and Child Center of the Chantal Biya Foundation (MCH/MCC-CBF) in Yaoundé, the Essos Hospital Center in Yaoundé (EHC) and the Laquintinie Hospital in Douala (LH)) under the coordination of the Centre Pasteur of Cameroon. The ANRS-PEDIACAM cohort study was designed to assess the feasibility of early HIV diagnosis and access of HIV-infected infants to antiretroviral multi-therapy and evaluate infant response to vaccines of the Expanded Program on Immunisation (EPI). A synthesis of the study protocol was described elsewhere [17]. Briefly, infant inclusion into the ANRS-PEDIACAM study was conducted in two consecutive phases. The first phase, planned from first week of life to 14 weeks, included all infants born live to HIV-infected mothers and an equivalent number of infants born to HIV uninfected mothers matched individually on gender and recruitment site. The newborn pairs were followed, tested for HIV (where needed) at the first follow-up visit planned at 6 weeks of age and given routine vaccinations. The samples were tested for HIV RNA by a real-time polymerase chain reaction (RT PCR) (Biocentric HIV Charge Virale) using the TaqMan technology in an in-house protocol validated by the French National Agency for Research on AIDS and Viral Hepatitis [18] or by Biocentric HIV DNA cell kit to test for HIV proviral DNA. In case of positive or indeterminate test result, a second test on a different sample was planned to confirm HIV infection [17]. For infants who tested HIV negative, a second was performed if the child became symptomatic or six week after weaning for breastfed infants. All identified HIV-infected infants and selected controls of uninfected infants followed from birth, born to HIV-infected mothers (HEU) or to HIV-uninfected mothers (HUU) were included in the second phase for prolonged follow up planned from 14 weeks. Inclusion into the second phase was also offered until October 2011 to HIV-infected children not identified during the first week of life but diagnosed before 7 months of age (HIL). After inclusion in the second phase, all HIV-infected infants were offered systematic cART according to Cameroonian guidelines. First line treatment was zidovudine (or stavudine for infant with anemia) and lamivudine associated with lopinavir/ritonavir if nevirapine (NVP) had been used for PMTCT, or with NVP otherwise. Follow-up was subsequently planned every 3 months for HIV-infected infants and every 6 months for HIV-uninfected infants, until the first 2 years. From 2 years, children were followed every 6 months until the last included child had 5 years [17]. Incentives, including free medical support for consultation, biological analysis, additional vaccines and reimbursement of transport costs, were provided to parents/caregivers by the project during follow up visits. The project provided during its first phase free milk as required to women who choose formula feeding.

### Participants and data collection

A case report form (CRF) was used to collect at the baseline and at different visits. This included data on the family environment (socio-demographic and economic characteristics of the family, information on parents), monitoring of pregnancy and childbirth (anthropometric parameters at birth, data concerning the PMTCT, clinical features of the infant) information on the anthropometric parameters of the children (weight, height, cranial and brachial perimeter). Weight was measure with the child supine and the crown of the head touching a vertical headboard using scale and flexible measuring tape respectively. For those aged 2 to 5 years, they step onto scale with one foot on each side. For the length, the child stands against the stadiometer on the board facing the practitioner Clinical status (clinical events since the last protocol visit), family environment, data related to ARV treatments received since the last visit (including adherence to treatment) was collected longitudinally. Biological data (blood count, lipid balance, viral load assessment (for HIV-infected children) and CD4 count were also collected.

### Main outcome definition and covariables

The main outcome was growth retardation including underweight, stunting or wasting defined at each visit using anthropometric indices as weight-for-age (WAZ) <-2, weight-for-length (WLZ) <-2, and length-for-age (LAZ) <-2 Z-scores respectively (7, 14). Z-score was calculated using the 2006 WHO growth standard [19].

Prior to the data analysis, a number of variables were recoded. Thus, the mother’s marital status was recoded in married, cohabitation and single/divorced/widow; the level of household income in <100,000 CFA francs and ≥100,000; the level of CD4 in <25% and ≥ 25%. The hemoglobin level was recoded according to the 2011 WHO criteria [20] as follows: not anemia (≥ 11 g / dl in children aged 0–24 months and ≥ 10 g / dl in in children aged 24–60 months), mild or moderate anemia (7 to 10.9 g / dl in children aged 0–24 months and 7–9.9 g / dl in children aged 24–60 months), and severe anemia (<7 g / dl). Sign of systemic involvement was defined at each visit by the presence of one or more of the following signs: fever, jaundice, hepatomegaly, adenopathy or parotitis. Chronic pathologies refers to a persistent pathologic condition observed or notified since the last visit (tuberculosis, sickle cell disease, malformations, chronic skin disease, etc.). Small for gestational age and gender (SGAG) defined as previously [21] as a birth weight Z-score adjusted for gestational age at delivery and gender that is more than two standard deviations below the mean (-2SD) and small birth size by any height Zscore < -2SD, in line with international recommendations [22].

### Statistical analysis

The Markov multi-state models were used to assess the incidence of growth retardation and identify associated factors. This choice was motivated by several constraints, including the presence of: (a) interval censorship with the moments and the rhythm of visits fixed a priori, (b) the informative censoring due to competing risk of malnutrition with death among children aged 0–5 years [3, 4, 9, 23–25], (c) the recurrence between healthy and malnourished states that need to be taken into account in the modeling process, (d) the episodic absences and loss to follow-up (LTFU) in the ANRS-PEDIACAM study, and (e) the presence of time-dependent variables.

We considered three states in our analysis: "healthy" (without malnutrition), "Malnourished" (when children presented growth retardation at the visit) and Death (for deceased children). Healthy and malnourished states were transient while the Death state was absorbent. Possible transitions between states were "Healthy—malnourished," "Healthy to Death," "Malnourished to Healthy" and "Malnourished—death". Such model is well described by Jackson [26] and implemented in the R package "msm". In order to combine multi-state models with multiple imputation, we implemented the algorithm for performing likelihood ratio tests with data from multiple imputation proposed by Xiao-Li [27, 28]. This model allowed us to investigate the factors associated with the instantaneous risk of underweight, stunting and wasting.

Growth patterns up to the age of 5 on the basis of WAZ, LAZ and WLZ was studied using the two-stage Heckman approach, whose generalization to repeated data has been proposed by Shelton *et al*. [29]. This method was chosen because its capacity to account for biases generated by missing data on the growth indices (dependent variables). Due to the participants’ behavior concerning LTFU, we hypothesize that the mechanism of the missing data was informative, placing us in the context of Missing Not At Random according to the missing data typology proposed by Rubin *et al*. [30].

In the first stage, a GEE probit model was used to identify factors associated with missing data at any visit of the scheduled follow-up, taking into account both the variables observed at baseline and time-dependent variables. The residuals of this model were used to compute the inverse mills ratio (IMR), representing the effect of all unobserved variables that can influence the missingness process. In the second stage, a GEE identity was used to model the marginal expectation of anthropometric indices. At this stage the IMR was added as a covariate to adjust for the bias due to the missing data in both univariable and multivariable analyses. In order to obtain valid hypothesis tests of the model parameters (p-values, standard errors and confidence intervals), we performed 500 bootstrap replications of each of the models adjusted with the IMR. Indeed, since the IMR has been estimated and not observed, the classical standard errors estimated in the model do not take into account the Heteroscedasticity induced by the selection of the sample [31], which invalidates the estimated standard errors.

In order to measure the influence of the biases due to the missing data on the conclusions made when they are not taken into account, we conducted an analysis using a standard GEE without taking into account the IMR. The results obtained with this sensitivity analysis were compared with those resulting from a procedure incorporating the bias correction.

The likelihood ratio test on multiple imputation data was carried out for the choice of the multi-state model variables, the Wald test for the GEE model and the choice based on a bootstrap procedure for the second step in the Heckman’s method. In order to take into account the potential confounding factors in the model, all variables with a p-value <0.25 were retained for the multivariable analysis. We followed a backward procedure for the selection of the variables to be maintained in the model. Before each variable was removed an investigation was done to take into account interaction terms. A variable was considered statistically significant if it had a p-value less than 0.05 or when the confidence interval of the associated estimate did not contain the value 0.

Due to the moderate proportion of missing around 10% accounting for all variables, we first imputed values using multiple imputations by conditional approach under the Missing At Random hypothesis. All variables (except anthropometric parameters) with missing data were included in the imputation model. This strategy for handling missing data was chosen because it allows both problems of bias and precision that could induce an inappropriate method to be resolved, especially when the imputation is done correctly [32]. We considered 10 multiple imputations with 10 iterations.

All analyzes were carried out using WHO Anthro (for the estimation of Z-scrores during follow-up) and R software, with the packages mice and mitools for multiple imputation, msm for multi-state model, geepack and BSagri for GEE.

## Results

### Study population description

**Fig 1:**
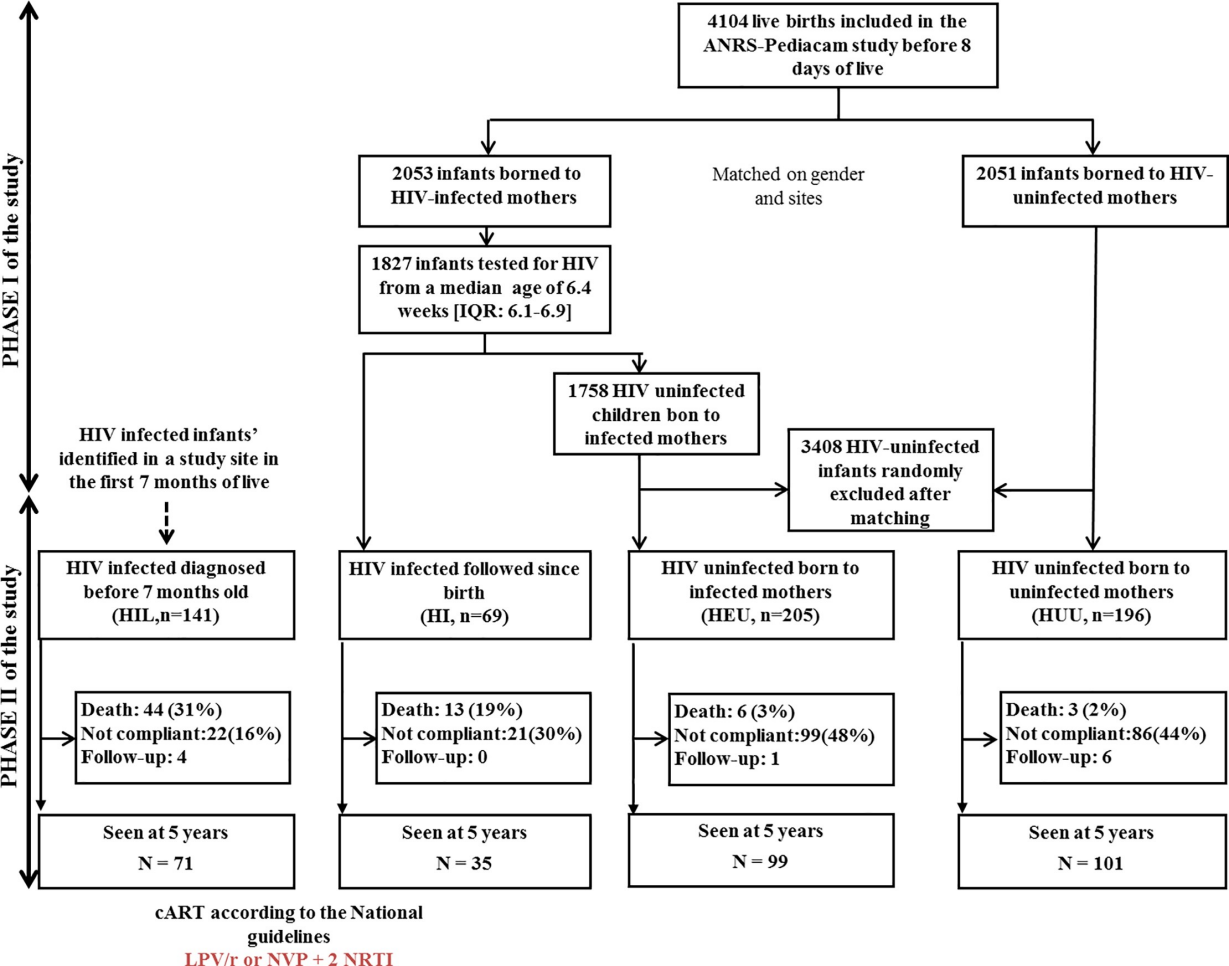
Brief synthesis of children included and followed until the age of 5, ANRS-PEDIACAM study, Cameroun, Nov. 2007- Dec.2015. IQR: interquartile range; Not compliant: failed to return for study visit over a period of one year; Follow-up: children regularly monitored, but under the age of 5 at the last visit; PHASE I: first study period planned the first week of life until 14 weeks; PHASE II: second study period for prolonged follow-up (three-monthly till 2 years and six-monthly till 5 years for HIV infected, six monthly till 5 years for HIV-uninfected).

Initially, a total of 4104 mother-child pairs were enrolled in the first phase of the ANRS-PEDIACAM study between November 2007 and October 2010, including 2051 HIV-exposed infants and 2053 HIV-unexposed infants. Among them, 1827 infants were tested for HIV at 6 weeks. For this analysis, 611 infants selected and included in the second phase of the ANRS-PEDIACAM study were enrolled for long-term follow-up, including 69 (11.3%) HI, 205 (33.5%) HEU, 196 (32.1%) HUU and 141 (23.1%) HIL, see **Fig 1**. Among the 210 children identify, 193 started cART at a median age of 4.1 months. A total of 124 were on LPV/r-based regimen while 69 were of NVP-based regimen. The median age at enrolment in the second phase of the study was 3.4 months (Interquartile range (IQR): 2.3–3.7) for HI, 4.3 months (IQR: 3.6–5.0) for HEU, 4.0 months (IQR: 3.6–4.9) for HUU, and 4.3 months (IQR: 3.2–5.6) for HIL. About 52.9% of children were girls. Nearly half of the children (48.9%) came from the Maternity of the Central hospital/Mother and Child Center of the Chantal Biya Foundation (MCH/MCC-CBF), 22.9% at the Laquintinie Hospital (LH) and 28.2% at the Essos Hospital Center (EHC). Concerning children's home environment, 56.6% and 54.5% of parents reported having water supply and a functional refrigerator at home, respectively. At birth, the proportion of small for gestational age and gender in HIV-infected infants was significantly higher than in HIV-uninfected infants (9.5% vs 5.2% p<10–2). Globally, the two groups of children differed significantly (**Table 1**).

**Table 1:** Comparison of the characteristics of mothers and children at the inclusion into the phase 2 of the ANRS-PEDIACAM study according to infant HIV status, Cameroun, Nov. 2007- Dec.2015.

	Inclusion group										
	HIV infected children				HIV uninfected children				Total		P
	HI		HIL		HEU		HUU				
	N	%	N	%	N	%	N	%	N	%	
**Mothers characteristics**	69	11.3	141	23.1	205	33.6	196	32.1	611	100	
Clinical site											
MCH/MCC-CBF *	30	43.5	76	53.9	98	47.8	95	48.5	299	48.9	0.40
LH ^†^	19	27.5	25	17.7	55	26.8	41	20.9	140	22.9	
EHC ^‡^	20	29.0	40	28.4	52	25.4	60	30.6	172	28.2	
Mother’s vital status											
Deceased	0	0.0	15	10.6	0	0.0	0	0.0	15	2.5	< .001
Mother’s marital status											
Married	44	63.8	85	60.3	135	65.9	133	67.9	397	65.0	0.87
Cohabitation	2	2.9	4	2.8	5	2.4	3	1.5	14	2.3	
Single/divorced/widow	23	33.3	50	35.5	65	31.7	60	30.6	198	32.4	
Mother’s level of education											
Higher education	8	11.6	6	4.3	43	21.0	84	42.9	141	23.1	< .001
Secondary	48	69.6	80	56.7	123	60.0	103	52.6	354	57.9	
Primary	12	17.4	53	37.6	37	18.0	8	4.1	110	18.0	
Electricity at home											
Yes	65	94.2	111	78.7	196	95.6	194	99.0	566	92.6	< .001
No	2	2.9	24	17.0	5	2.4	0	0.0	31	5.1	
Water supply at home											
Yes	36	52.2	63	44.7	106	51.7	141	71.9	346	56.6	< .001
No	31	44.9	72	51.1	95	46.3	51	26.0	249	40.8	
Functional fridge at home											
Yes	34	49.3	53	37.6	108	52.7	138	70.4	333	54.5	< .001
No	32	46.4	80	56.7	92	44.9	53	27.0	257	42.1	
**Children characteristics**											
Sex											
Female	42	60.9	75	53.2	108	52.7	98	50.0	323	52.9	0.49
SGAG at birth ^$^											
Yes	13	18.8	7	5.0	17	8.3	4	2.0	41	6.7	< .001
No	56	81.2	101	71.6	188	91.7	191	97.4	536	87.7	
Small size at birth											
Yes	4	5.8	3	2.1	13	6.3	1	0.5	21	3.4	< .001
No	57	82.6	36	25.5	189	92.2	191	97.4	473	77.4	
Hospitalisation											
Yes	14	20.3	78	55.3	10	4.9	4	2.0	106	17.3	< .001
No	52	75.4	63	44.7	195	95.1	192	98.0	502	82.2	
Chronic pathologies											
Yes	0	0.0	12	8.5	0	0.0	2	1.0	14	2.3	< .001
No	65	94.2	106	75.2	205	100.0	194	99.0	570	93.3	
Diarrhea											
Yes	9	13.0	55	39.0	8	3.9	8	4.1	80	13.1	< .001
No	55	79.7	83	58.9	197	96.1	188	95.9	523	85.6	
CD4 (in %)											
< 25	30	43.5	95	67.4	15	7.3	28	14.3	168	27.5	< .001
≥ 25	34	49.3	43	30.5	185	90.2	156	79.6	418	68.4	
Anemia											
Mild	18	26.1	24	17.0	18	8.8	25	12.8	85	13.9	< .001
Moderate / Severe	16	23.2	58	41.1	8	3.9	10	5.1	92	15.1	
No	31	44.9	56	39.7	177	86.3	149	76.0	413	67.6	
Status											
Compliant	35	50.7	75	53.2	100	48.8	107	54.6	317	51.9	< .001
Deceased	13	18.8	44	31.2	6	2.9	3	1.5	66	10.8	
Not compliant	21	30.4	22	15.6	99	48.3	86	43.9	228	37.3	

HI: HIV infected followed since birth; HIL: HIV infected diagnosed before 7 months old; HEU: HIV uninfected born to infected mothers; HUU: HIV uninfected born to uninfected mothers

*MCH/MCC-CBF = Maternity of the Central hospital/Mother and Child Center of the Chantal Biya Foundation

^†^ LH = Laquintinie Hospital

^‡^ EHC = Essos Hospital Center

^$^ SGAG = Small for Gestational Age and Gender; Compliant: children regularly monitored; Chronic pathologies: included chronic diseases or malformations observed and notified by the clinician since the last visit; Anemia: obtained from the level of hemoglobin, according to the 2011 WHO criteria; Not compliant: lost-to-follow-up for over a year before 5 years.

From enrolment until 60 months, 48% of infants were lost-to-follow up (not compliant) in HEU, 44% in HUU, 30% in HI, and 16% in HIL. In total, 10.8% of infants (n = 66) died during the same period including 18.8% (n = 13), 2.9% (n = 6), 1.5% (n = 3) and 31.2% (n = 44) respectively from HI, HEU, HUU, and HIL children groups. The median age at death was not statistically different between groups (6.7 months (IQR = 3.2–12.2) among HI, 6.2 (IQR = 4.0–9.2) among HIL, 16.6 (IQR = 6.6–41.8) among HEU, and 30.6 (IQR = 17.9–47.5) among HUU; p = 0.14). Also, median follow-up time at the last visit was 60 months (IQR = 41.0–60.2) among HI, 59.8 months (IQR = 11.7–60.2) among HIL, 59.6 months (IQR = 33.8–60.3) among HEU, and 60.0 months (IQR = 42.6–60.4) among HUU. Among alive children, 2.0% (11/545) were less than 5 years at the last visit, with a median age of 54.6 months (range 54.3–55.4) (**Fig 1**). Overall, 61%, 42.3%, and 27.7% of children experienced wasting, stunting, and underweight at least once respectively with significantly high proportions in HIL group. Regardless of the indicator of malnutrition used, there was a high proportion of malnutrition among children from HIL group, followed respectively by children from HI, HEU and HUU groups (**Table 2**). Among children who died, 63.3% were stunted (38/60), 51.6% (32/62) were underweight, and 26.7% (16/60) wasted.

**Table 2:** Distribution of children according to whether they have undergone at least one or not malnutrition during follow-up in the ANRS-PEDIACAM study, Cameroun, Nov. 2007- Dec.2015.

	Inclusion group								Total		
	HI		HIL		HEU		HUU				P
	n	%	n	%	n	%	n	%	N	%	
WAZ < -2SD (n = 607)											
Yes	24	36.9	88	62.4	41	20.0	15	7.7	168	27.7	0.004
LAZ < -2SD (n = 605)											
Yes	47	72.3	115	82.7	124	60.5	83	42.3	369	61.0	0.071
WLZ < -2SD (n = 605)											
Yes	32	49.2	68	48.9	91	44.4	65	33.2	256	42.3	0.403

HI: HIV infected followed since birth; HIL: HIV infected diagnosed before 7 months old; HEU: HIV uninfected born to infected mothers; HUU: HIV uninfected born to uninfected mothers; WAZ: Weight-for-age Zscore; LAZ: Length-for-age Zscroe; WLZ: Weight-for-length Zscore; SD: standard deviation.

**Fig 2:**
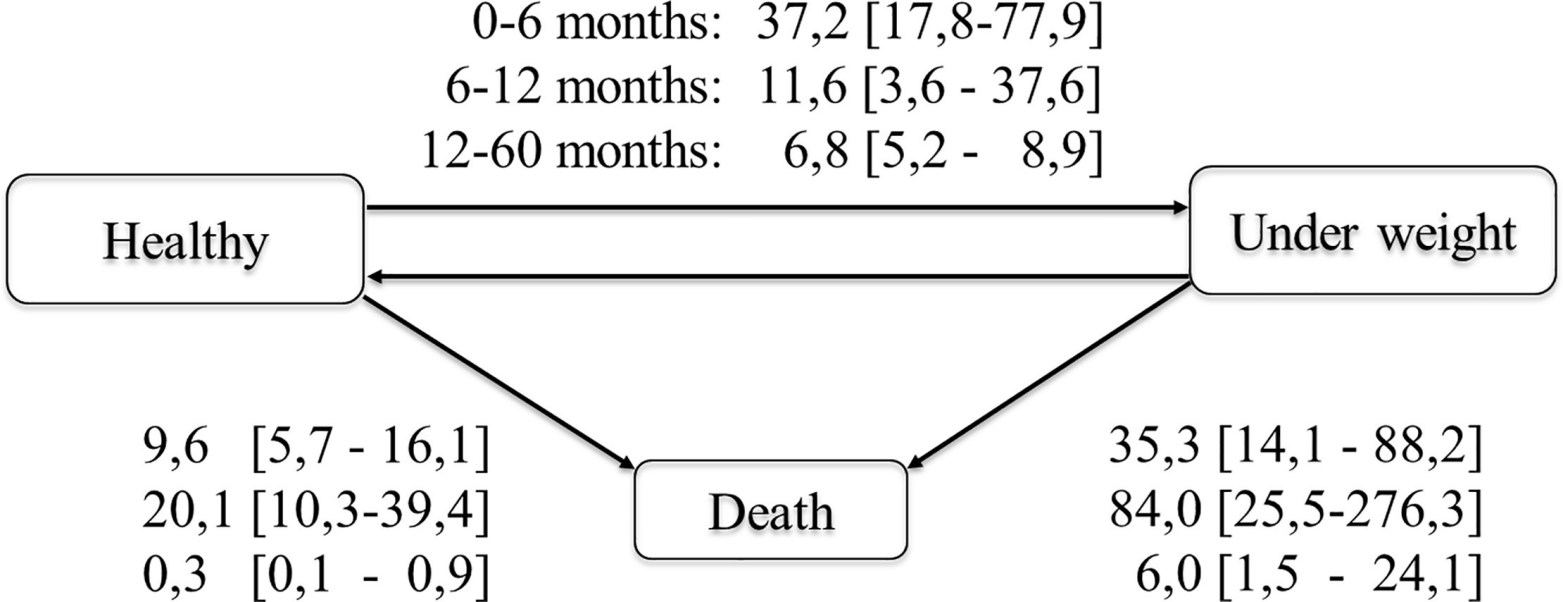
Transitions intensities per 1000 person-months between healthy malnutrition and death status for underweight, ANRS-PEDIACAM study, Cameroun, Nov. 2007- Dec.2015.

The transitions intensity between healthy status to underweight estimated from the multi-state model were 37.2 per 1000 person-months (95% Confidence interval (95%CI): 17.8–77.9) between 0–6 months, 11.6 per 1000 person-months (95%CI: 3.6–37.6) between 6–12 months, and 6.8 per 1000 person-months (95%CI: 5.2–8.9) between 12–60 months. Between healthy to death, it was 9.6 (95%CI: 5.7–16.1), 20.1 (95%CI: 10.3–39.4), and 0.3 (95%CI: 0.1–0.9) per 1000 person-months between 0–6 months, 6–12 months and 12–60 months respectively. Between underweight to death status, it was 35.3 (95%CI: 14.1–88.2), 84.0 (95%CI: 25.5–276.3) and 6.0 (95%CI: 1.5–24.1) per 1000 person-months between 0–6 months, 6–12 months and 12–60 months respectively. Globally, the incidence of growth retardation was higher in HIV-infected children (HI and HIL) than in uninfected children (HEU and HUU) regardless of the indicator of malnutrition considered (see **Fig 2**).

### Factors associated with growth retardation

In multivariable multistate analysis, children’s group, children’s age, and occurrence of chronic pathologies since last visited were independently associated with wasting (see **Table 3**). At any particular time during follow-up, HI children experienced wasting more frequently compared to HEU (adjusted HR (aHR): 3.3 (95%CI: 1.4–7.9) regarding the HI children and aHR: 4.3 (95%CI: 1.9–9.8) regarding the HIL children. HEU and HUU groups were comparable (aHR: 0.7 (95%IC = 0.3–1.5)). The most at risk age group was 0–6 months compared to 6–12 months with an aHR of 8.8 (CI = 3.4–23.2). Children with chronic pathologies since the last visit were 6.7 times more likely to be wasted (CI: 3.7–12.3). HIL and HI children died frequently from healthy status compared to HEU children (aHR = 14.8[4.1–53.0] 5.8[1.5–22.3] respectively); compared to children aged 6–12 months, the aHR of death was 2.2[1.1–4.7] and 0.0[0.0–0.2] in children aged 0–6 months and 12–60 months resp. Recovery after wasting was explained by children’s group (high odds in HI compared to HEU), young age (0–6 months compared to 6–12 months), male gender, non-hospitalization before inclusion and the absence of general signs at the visit (**Table 3**).

**Table 3:** Multivariable multistate models and factors associated with the risk of wasting (WLZ <-2SD) and underweight (WAZ <- 2SD) in the ANRS-PEDIACAM study, Cameroun, Nov. 2007- Dec.2015.

	Transitions											
	Healthy- malnourished				Healthy-death				Malnourished-Healthy			
	aHR	L	U	P	aHR	L	U	P	aHR	L	U	P
**Factors associated with wasting (WLZ < - 2SD)**												
Children’s groupe (ref = HEU)												
HI	3.3	1.4	7.9	0.01	5.8	1.5	22.3	0.01	3.3	1.4	7.8	0.01
HIL	4.3	1.9	9.8	< .001	14.8	4.1	53.0	< .001	4.4	2.0	9.5	< .001
HUU	0.7	0.3	1.5	0.31	0.2	0.0	4.8	0.35	1.0	0.5	1.9	0.96
Children’s age (months, ref = (6–12])												
(0–6]	8.8	3.4	23.2	< .001	2.2	1.1	4.7	0.03	5.3	2.4	12.0	< .001
(12–60]	0.8	0.4	1.7	0.60	0.0	0.0	0.2	< .001	0.7	0.4	1.3	0.22
Gender: Female	0.7	0.4	1.2	0.26	1.4	0.8	2.7	0.26	0.6	0.3	0.9	0.02
Hospitalisation before inclusion	1.1	0.6	2.1	0.83	1.9	0.9	3.7	0.07	0.3	0.2	0.6	< .001
General signs during the visit									0.5	0.3	0.9	0.03
Chronic pathologies	6.7	3.7	12.3	< .001								
**Factors associated with underweight(WAZ < - 2SD)**												
Children’s groupe (ref = HI)												
HEU	1.4*	0.6	3.2	0.43	0.2	0.0	0.8	0.03	1.9	1.0	3.5	0.04
HIL	2.2*	1.0	4.9	0.05	1.4	0.5	4.4	0.53	1.6	0.9	2.8	0.12
HUU	0.3*	0.1	1.0	0.04	0.1	0.0	0.6	0.01	1.8	0.8	4.0	0.15
HEU: CD4 <25%	0.2	0.0	2.6	0.24								
HIL : CD4 <25%	1.2	0.2	6.9	0.87								
HUU: CD4 <25%	0.0	0.0	Inf	0.53								
CD4 (ref = ≥ 25%)												
< 25%	3.0^†^	1.3	7.3	0.01					0.7	0.5	1.1	0.14
< 25%: HEU	0.5	0.0	6.1	0.58								
< 25%: HIL	1.6	0.3	10.3	0.61								
< 25%: HUU	0.0	0.0	Inf	0.68								
Children’s age (months, ref = (6–12])												
(0–6]	2.7	1.4	5.2	0.00	1.3	0.3	4.9	0.74	1.8	1.0	3.1	0.04
(12–60]	0.3	0.2	0.5	< .001	0.2	0.1	0.8	0.02	0.6	0.4	1.0	0.07
Sex: Female	0.5	0.4	0.8	0.00	2.4	0.8	6.8	0.11	0.6	0.4	0.9	0.01
Professional activity of the mother (ref = paid activity)												
Training/student	0.6	0.3	1.2	0.14					0.4	0.2	0.8	0.01
Housewife/unemployed	0.9	0.6	1.5	0.73					0.6	0.4	0.9	0.01
Water supply at home	0.7	0.5	1.1	0.17					1.5	1.0	2.2	0.04
Hospitalisation before inclusion	1.9	1.2	2.9	0.01	0.8	0.3	2.4	0.74	0.6	0.4	0.9	0.02
Chronic pathologies	2.2	0.9	5.5	0.09					0.3	0.1	0.8	0.02

*In children’ with more than 25% of CD4

^†^in HIV-infected infants; HI: HIV infected followed since birth; HIL: HIV infected diagnosed before 7 months old; HEU: HIV uninfected born to infected mothers; HUU: HIV uninfected born to uninfected mothers; WAZ: Weight-for-age Zscore; WLZ: Weight-for-length Zscore; SD: standard deviation; aHR: adjusted hazard risk; L: Lower bound of the confidence interval; U: Upper bound of the confidence interval; P: Pvalue; Inf: Value greater than 100.

Concerning factors associated with underweight (see **Table 3**), we identified children’s group, mainly in those with more than 25% of CD4, low CD4 count, children’s age, gender, and hospitalization before inclusion. In children with more than 25% CD4 at the last visit, compared to HI children, the aHR was 2.2 (95%CI: 1.0–4.9) among HIL children and 0.3 (95%CI: 0.1–1.0) in HUU ones. Among HI children, those with less than 25% CD4 were more likely to develop underweight than those with CD4 count > 25% (aHR: 3.0, 95%CI: 1.3–7.3). Compared to the 6–12 months age group, children aged 0–6 months were 2.7 more likely to develop underweight (95%CI: 1.4–5.2) while children aged 12–60 months were protected (aHR: 0.3, 95%CI: 0.2–0.5), female gender were protected (aHR: 0.5, 95%CI: 0.4–0.8). Hospitalized prior to inclusion presented a risk of 1.9 (95%CI: 1.2–2.9). Although, not statistical significant, children with chronic pathologies since the last visit had a risk of 2.2 (95%CI: 0.9–5.5). “Healthy-death” transition was associated with children group (HEU and HUU protected compared to HI children) and age (children aged 12–60 months protected compared to those aged 6–12 months). Factors independently associated with the odds of recover after underweight were children’s age, the male gender, mothers’ professional activity (more observed in mothers with paid activity), presence of running water at home, absence of hospitalization before inclusion, and absence of chronic pathology since the last visit (see **Table 3**).

Associated factors with stunting, as show in **Table 4** were children’s group, clinical site, age (children aged 0–6 months were at risk and children aged 12–60 months protected compared to those aged 6–12 months), small size at birth (aHR: 2.0, 95%CI: 1.1–3.6), diarrhea (aHR: 1.8, 95%CI: 1.2–2.8), the low level of CD4 count (aHR: 1.4, 95%CI: 1.1–1.8). In the MCH/MCC-CBF, compared to HEU children, the adjusted hazard risk of stunting was 8.4 (95%CI: 2.4–19.7) in HIL children, 1.5 (95%CI: 0.9–2, 5) in HI children and 0.8 (95%CI: 0.5–1.2) in HUU. Compared to HEU children, the risks were comparable as well in LH as in EHC with HI, HIL and HUU children. On the other hand, compared to children included in the MCH/MCC-CBF, the aHR of stunting was respectively 2.4 (95%CI: 1.5–3.8), 2.8 (95%CI: 0.8–9.6), 0.1 (95%CI: 0.0–0.7) and 3.8 (95%CI: 1.3–11.1) in the LH among HEU, HI, HIL, and HUU children. Although not statistical significant, the presence of water supply at home was a protective factor of stunting (aHR: 0.8, 95%CI: 0.6–1.0). The presence of systemic clinical signs at the visit was associated with death from healthy state (aHR = 11.0, CI = 1.4–88.0). Recovery after stunting was associated with children group (HUU compared to HEU), clinical site (LH compared to MCH/MCC-CBF), age of the child (0–6 months compared to 6–12 months) and the absence of chronic pathologies since the last visit.

**Table 4:** Multivariable multistate models and factors associated with the risk of stunting (LAZ <-2SD) in the ANRS-PEDIACAM study, Cameroun, Nov. 2007- Dec.2015.

	Transitions											
	Healthy- malnourished				Healthy-death				Malnourished-Healthy			
	aHR	L	U	P	aHR	L	U	P	aHR	L	U	P
Children’s group (ref = HEU)												
HI	1.5*	0.9	2.5	0.12					0.9	0.6	1.3	0.64
HIL	8.4*	2.4	29.7	0.00					0.8	0.5	1.3	0.32
HUU	0.8*	0.5	1.2	0.27					1.5	1.0	2.2	0.03
HI: LH	1.8	0.5	6.3	0.38								
HIL: LH	0.4	0.0	5.7	0.53								
HUU: LH	1.2	0.4	3.6	0.79								
HI: EHC	1.9	0.5	6.9	0.31								
HIL: EHC	1.5	0.1	19.6	0.77								
HUU: EHC	0.9	0.3	3.1	0.89								
Clinical site (ref = MCH/MCC-CBF)											
LH	2.4^†^	1.5	3.8	< .001	0.5	0.1	5.3	0.59	0.6	0.4	0.9	0.01
EHC	1.0^†^	0.6	1.6	0.86	0.4	0.0	122.1	0.75	1.2	0.8	1.7	0.36
LH: HI	2.8	0.8	9.6	0.09								
EHC: HI	1.2	0.3	4.3	0.76								
LH: HIL	0.1	0.0	0.7	0.02								
EHC: HIL	0.2	0.0	1.0	0.06								
LH: HUU	3.8	1.3	11.1	0.02								
EHC: HUU	1.2	0.3	4.0	0.83								
Children’s age (months, ref = (6–12])											
(0–6]	1.6	1.0	2.5	0.06					2.5	1.5	4.4	0.00
(12–60]	0.2	0.1	0.3	< .001					0.9	0.6	1.5	0.81
Water supply at home	0.8	0.6	1.0	0.07	0.2	0.0	2.4	0.23	1.6	0.9	3.0	0.14
Small size at birth	2.0	1.1	3.6	0.03					1.5	0.9	2.5	0.13
General signs during the visit	1.2	0.8	1.7	0.41	11.0	1.4	88.0	0.02				
Diarrhea since the latest visit	1.8	1.2	2.8	0.01								
Chronic pathologies									0.4	0.1	0.9	0.03
CD4 < 25%	1.4	1.1	1.8	0.02								

*In children’ from the Maternity of the Central hospital/Mother and Child Center of the Chantal Biya Foundation

^†^in HIV-exposed uninfected children; HI: HIV infected followed since birth; HIL: HIV infected diagnosed before 7 months old; HEU: HIV uninfected born to infected mothers; HUU: HIV uninfected born to uninfected mothers; MCH/MCC-CBF: Maternity of the Central hospital/Mother and Child Center of the Chantal Biya Foundation; LH: Laquintinie Hospital; EHC: Essos Hospital Center; LAZ: Length-for-age Zscore; SD: standard deviation; aHR: adjusted hazard risk; L: Lower bound of the confidence interval; U: Upper bound of the confidence interval; P: Pvalue.

### Factors associated with growth patterns up to the age of 5

**Fig 3:**
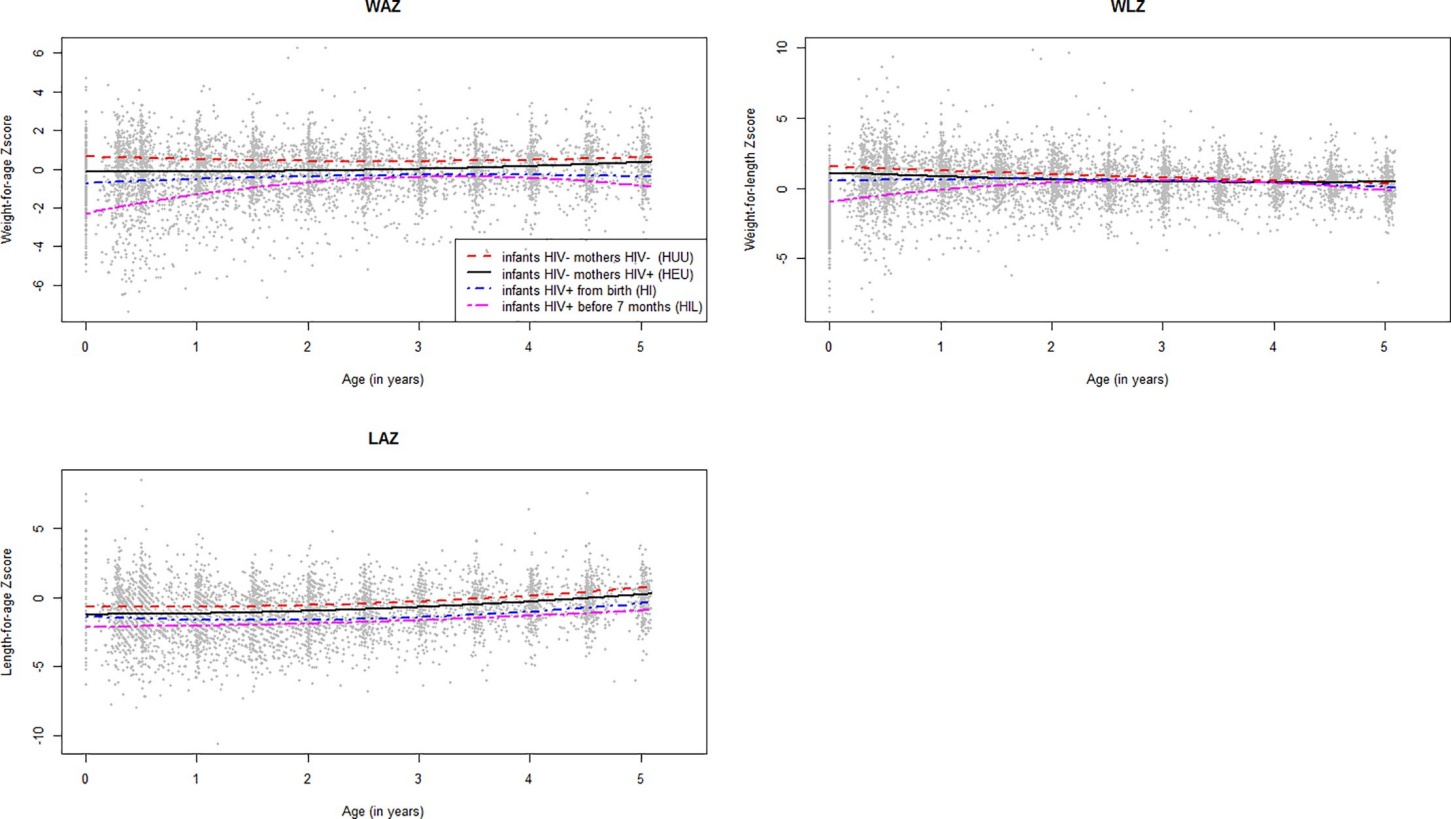
Mean growth of children according to anthropometric index and infants’ group at inclusion, from a second order polynomial regression model including 2775 observations for WAZ, 2763 observations for LAZ, and 2758 observations for WLZ, ANRS-PEDIACAM study, Cameroun, Nov. 2007- Dec.2015.

**Fig 3** presents the average evolution of the anthropometric indices of children followed in the ANRS-PEDIACAM study according to the different groups. For weight-for-age Z-score (WAZ), mean growth of HIL children was delayed compared to children in other groups, especially in the first two years of life. For the weight-for-length Z-score (WLZ), from 24 months of age, we observed an identical mean evolution in all groups. We will not present results from univariable analyze and Heckman’s first-stage models explaining the non-response process.

In Multivariable analysis of the WLZ evolution, two significant interactions were observed between children’s group with the onset of an infectious pathology, and with anemia **Table 5**. A mean reduction of WLZ in HIL children in the presence of anemia and infectious pathology compared to HEU children. Moreover, the onset of infectious pathologies in HEU children reduced the WLZ by nearly 14%. Other variables such as female gender, multiple birth, reported developmental delay, presence of systemic clinical signs, chronic pathologies since last visit, low birth weight (SGAG), and mother’s primary level of education were independently associated to the reduction of the mean WLZ evolution relative to the reference class (coefficient <0 and P <0.05, see **Table 5**). By cons, changes of residence, breastfeeding, and the presence of electricity were independently associated to an increase of the average WLZ evolution compared to the reference class.

**Table 5:** Multivariable model, GEE identity with exchangeable correlation structure, describing the factors associated with the evolution of WLZ in the ANRS-PEDIACAM cohort (with and without taking into account biases due to missing data on the WLZ), Cameroun, Nov. 2007- Dec.2015.

	Multivariable analysis without IMR N = 4919 visits by 605 children						Multivariable analysis with IMR: SE, L, U et P obtained by bootstrap*				
	Coef	SE		L	U	P	Coef	SE	L	U	P
**IMR**	** **	** **		** **	** **	** **	**-0.69**	**0.239**	**-1.13**	**-0.18**	**0.002**
Clinical site (ref = MCH/MCC-CBF)											
LH	0.90	0.106		0.69	1.11	< .001	0.94	0.078	0.79	1.09	< .001
EHC	0.10	0.088		-0.07	0.27	0.257	0.04	0.071	-0.09	0.19	0.546
Sex: Female	-0.21	0.077		-0.36	-0.06	0.007	-0.16	0.061	-0.28	-0.04	0.010
Age	0.84	0.056		0.73	0.95	< .001	0.96	0.063	0.83	1.08	< .001
Age^2^	-0.16	0.010		-0.18	-0.14	< .001	-0.17	0.008	-0.18	-0.15	< .001
**Multiple birth**	**-0.39**	**0.223**		**-0.83**	**0.04**	**0.077**	**-0.40**	**0.181**	**-0.78**	**-0.09**	**0.030**
Children’s groupe (ref = HEU)	** **	** **		** **	** **	** **					
**HI**	**0.08**	**0.137**	**-0.19**		**0.35**	**0.564**	**-0.08**	**0.118**	**-0.30**	**0.15**	**0.524**
**HIL**	**0.05**	**0.135**	**-0.21**		**0.32**	**0.701**	**-0.17**	**0.135**	**-0.41**	**0.11**	**0.216**
**HUU**	**0.27**	**0.115**	**0.04**		**0.49**	**0.020**	**0.17**	**0.094**	**-0.01**	**0.35**	**0.082**
Infectious patologies (ref = No)	-0.14	0.066		-0.27	-0.01	0.038	-0.14	0.048	-0.23	-0.05	0.002
Anemia (ref = No)	0.06	0.088		-0.11	0.24	0.469	0.07	0.063	-0.06	0.18	0.302
HI: Infectious patologies	-0.17	0.126		-0.42	0.07	0.167	-0.17	0.101	-0.37	0.05	0.090
HIL: Infectious patologies	-0.29	0.105		-0.49	-0.08	0.006	-0.28	0.084	-0.44	-0.10	< .001
HUU: Infectious patologies	0.02	0.094		-0.16	0.21	0.807	0.03	0.073	-0.12	0.17	0.680
HI: Anemia	-0.02	0.172		-0.36	0.32	0.899	-0.07	0.128	-0.34	0.17	0.586
HIL: Anemia	-0.47	0.128		-0.72	-0.21	< .001	-0.38	0.105	-0.59	-0.17	< .001
HUU: Anemia	-0.15	0.130		-0.40	0.11	0.258	-0.12	0.101	-0.32	0.07	0.238
**CD4 ≥_25%**	**0.13**	**0.069**		**-0.01**	**0.26**	**0.063**	**0.04**	**0.064**	**-0.08**	**0.17**	**0.528**
Home change	0.17	0.072		0.03	0.32	0.016	0.20	0.050	0.10	0.30	< .001
Developmental delay	-0.68	0.131		-0.93	-0.42	< .001	-0.67	0.102	-0.87	-0.48	< .001
**General signs during the visit**	**-0.14**	**0.087**		**-0.31**	**0.03**	**0.107**	**-0.14**	**0.067**	**-0.27**	**-0.01**	**0.032**
**Breastfeeding**	**0.06**	**0.072**		**-0.08**	**0.20**	**0.391**	**0.21**	**0.081**	**0.04**	**0.35**	**0.016**
**Diarrhea**	**-0.27**	**0.105**		**-0.48**	**-0.07**	**0.010**	**-0.15**	**0.091**	**-0.35**	**0.02**	**0.096**
Chronic pathologies	-0.63	0.175		-0.97	-0.29	< .001	-0.63	0.138	-0.87	-0.32	< .001
SGAG (ref = No)	-0.46	0.169		-0.79	-0.13	0.006	-0.51	0.125	-0.77	-0.27	< .001
**Mother’s level of education (ref = higher)**					** **	** **					
**Secondary**	**-0.01**	**0.091**		**-0.19**	**0.17**	**0.882**	**0.02**	**0.069**	**-0.11**	**0.15**	**0.808**
**Primary**	**-0.24**	**0.136**		**-0.51**	**0.03**	**0.080**	**-0.28**	**0.111**	**-0.47**	**-0.04**	**0.016**
**Electricity supply at home**	**0.34**	**0.238**		**-0.13**	**0.81**	**0.152**	**0.35**	**0.183**	**0.02**	**0.74**	**0.052**

*standard errors (SE) and confidence intervalle (L,U) obtained based on 500 replications bootstrap, due to the introduction of the IMR in the model; IMR: Inverse Mills ratio obtained from the residuals of the first stage model; Coef: coefficients; L: Lower bound of the confidence interval; U: Upper bound of the confidence interval; P: Pvalue; HI: HIV infected followed since birth; HIL: HIV infected diagnosed before 7 months old; HEU: HIV uninfected born to infected mothers; HUU: HIV uninfected born to uninfected mothers; MCH/MCC-CBF: Maternity of the Central hospital/Mother and Child Center of the Chantal Biya Foundation; LH: Laquintinie Hospital; EHC: Essos Hospital Center; SGAG: small-for-gestational age and gender; WLZ: Weight-for-length.

Concerning the mean evolution of WAZ, HIV-infected children contributed negatively while the HUU children's contribution was positive compared to HEU children (P <0.001, see **S1 Table**). In addition, the following mother’s and child’s characteristics impacted positively (breastfeeding, the presence of electricity and water at home) or negatively (multiple births, chronic pathologies, anemia, CD4 count < 25%, unemployed mother, developmental delay, presence of systemic clinical signs during the visit, SGAG, diarrhea, secondary and primary education level of the mother) WAZ evolution.

Similar observation was made at with LAZ (see **S2 Table**). We also observed a significant interaction with positive coefficients between the children group and anemia, showing that the negative effects of HI and HIL children (respectively positive effects of HUU children) compared to HEU children on LAZ were attenuated (respectively accentuated) in the presence of anemia. Furthermore, in children, the presence of anemia contributes negatively to the evolution of WAZ. It was also observed that children with small birth size contribute negatively to the LAZ evolution.

A mean quadratic effect of age was observed on WLZ, WAZ or LAZ profiles. Taking into consideration the selection bias of missing data, the estimated IMR effect was significantly less than 0 with WLZ and WAZ profiles and some coefficients of the standard GEE models have changed their significance (see **Table 5** and **S1 Table**). No significant effect of IMR was observed on the LAZ evolution (see **S2 Table**).

## Discussion

The main focus of this analysis was to compare incidence of growth retardation, instantaneous risk of death related to growth retardation, and growth evolution over the first five years of life between early treated HIV-infected, HEU and HUU children in Cameroon. Other studies [13, 14] also addressed the issue of growth among children in the context of early cART in sub-Saharan Africa. But, the distinctiveness of our study was its statistical approach, which considered deaths as a competitive risk with malnutrition, and the recurrence of malnutrition during follow-up.

Overall, our results showed that, regardless of the anthropometric index considered, early treated HIV-infected children (particularly those from HIL group) had growth retardation and average growth rate lower than that of HIV-uninfected children indicating no catching up of growth indices over the first five years of life. It is known that administration of early ART helps to suppress viral load and improve the immune response leading drastic reduction of morbidity, mortality [4]. These observations can be the consequences of the differences in characteristics of early ART treated HIV-infected children and HIV-uninfected children. As presented in Table 1, early HIV-infected children environment is socioeconomically disadvantage (low education level of mothers, low access to electricity and water etc.) compared to HIV-uninfected children. Furthermore, a significant number of early treated HIV-infected children will fail to achieve or to maintain virological suppression. Thus, several factors can influence the response to antiretroviral therapy, and therefore the nutritional response. Even with a significant gain in weight and height in presence of cART, the nutritional status could not be completely restored for a significant number of children who remained malnourished, despite the treatment [33]. ART alone is not sufficient and specific interventions are needed to improve the nutritional care of HIV-infected children.

In the HIL group, a high proportion of deaths occurred during the first year of life corroborating the fact that without cART, one third of HIV-infected children died before the age of one year [34]. This was probably due to advanced stage of the disease or CMV coinfection as recently described at time of enrolment [35]. Many authors have also reported that malnourished children died in average 2 to 3 times in the first month of cART treatment compared to non-malnourished children [9, 23–25].

Some authors have reported the negative effect of HIV infection on growth in different settings [36, 37]. Bailey *et al*. in Congo found in a prospective cohort of children that, the risk of underweight, stunting and wasting was higher in HIV-infected children than in HIV-uninfected children during the first two years of life [36]. In Rwanda, in a cohort of children aged 0–48 months, HIV-infected children were more likely to be underweight and stunting, but not wasting compared to HIV-uninfected children. In that cohort, comparable anthropometric indices were observed between HEU and HUU [37]. HIV-infected children suffer from opportunistic infections, such as diarrhea and persistent malabsorption, with inflammatory effects that can cause intestinal dysfunction and affect height and weight [38].

HUU children had a risk of wasting and stunting comparable to that of HEU. Furthermore, in children with CD4 levels greater than 25%, those in HUU group were protected against underweight compared to those in HI group. However, regardless of the anthropometric index considered, the average growth evolution was negatively impacted by HIV infection and in-utero HIV exposition. This requires special attention to be given to children born to HIV-infected mothers to ensure growth comparable to that of HUU. Nutritional supplements during the first years of life observed by Rebacca *et al*. as in many other studies indicates that such action in this period would improve the growth of HIV-exposed children [39].

Many other factors, such as male gender, small birth size, multiple birth, mother's primary or secondary education, SGAG, lack of running water or electricity at home, unemployment of the mother have been identified as potentially and independently compromising the growth of children in this study. In addition, others factors related to the child's health status such as the occurrence of chronic pathologies, reported episodes of diarrhea, anemia, and low CD4 levels affecting the growth of children have also been identified in other contexts [40].

In the modeling process, we found in some models, mainly for the variable children’s group fairly broad confidence intervals. This is due to the low number of events observed in HUU children during follow-up. The interaction was significant between the groups of children and the clinical site, reflecting the heterogeneity in the management of the children. The study sites were located in referral hospital, pioneers and pole of excellence in pediatric HIV care in Cameroon, managing the largest number of HIV-infected and HIV exposed children.

Heckman's method allowed us to avoid falsely concluding on the effect (or not) of different variables on the evolution of anthropometric indices as reported by Protopopescu et al [31]. This analysis approach is necessary in our context where it is not obvious to involve HIV-uninfected children’s parents (infected or not) to research studies involving HIV-infected children and with no direct benefit.

The multi-state models of the Markovian type used allowed us to study the dynamics evolution of the growth retardation taking into account the censorship interval observed on the occurrence of the growth retardation. Available studies are mostly based on Cox proportional hazard models that consider right censorship of deaths and first occurrence of growth retardation. Another interesting aspect of this study was the missing data consideration using the most well-known methods for minimizing induced biases based on the hypothesis of the non-response process: multiple imputation and Heckman methods.

Three common growth indicators encountered during the first five years of live in the literature were used: weight-for-age, weight for length, and length-for-age Z-score. One of the perspectives of this work would be to define, using these 3 indices, a single indicator for the growth retardation. We also plan to propose a user friendly R package, which summarizes all the statistical methods used in this manuscript, for wide use by the scientific community. A comparative analysis between the Markov multi-state model and the joint frailty model for recurrent events and death [41] should be considered to find the most appropriate method for studying the incidence of growth retardation and the instantaneous risk of death. The advantage of the latter method (Rondeau et al) is to take into account the potential selection biases due to unmeasured covariates, using a shared frailty term.

## Conclusions

Growth retardation in the ANRS-PEDIACAM study was identified as a real health problem affecting at least once more than 60% of children followed during the first 5 years of life in three referral hospitals based in urban setting in Cameroon. HIV-infected children diagnosed before the age of 7 months were most affected, especially during the first two years of life, where their mean growth evolution diverged drastically from that of other groups. Uninfected infants born to HIV-infected mothers were comparable to un-exposed children in terms of the instantaneous risk of growth retardation. Overall, mother’s or child HIV infection affects the child's growth during the first years of life, regardless of the availability of antiretroviral therapy. However, it is imperative to strengthen the care of HIV-infected children and to follow international guidelines with regard to the early detection and initiation of ARVs. Special attention should be given to the statistical methodology envisaged for the study of the growth of children in order to take into account the potential biases due to missing data and other constraints related to the epidemiological problem.

## Supporting information

S1 Table Multivariable model, GEE identity with exchangeable correlation structure, describing the factors associated with the evolution of WAZ in the ANRS-PEDIACAM cohort (with and without taking into account biases due to missing data on the WAZ), Cameroun, Nov. 2007- Dec.2015. (DOCX)

S2 Table Multivariable model, GEE identity with exchangeable correlation structure, describing the factors associated with the evolution of LAZ in the ANRS-PEDIACAM cohort (with and without taking into account biases due to missing data on the LAZ), Cameroun, Nov. 2007- Dec.2015. (DOCX)
