# Supplementary material for: Early treated HIV-infected children remain at risk of growth retardation during the first five years of life: Results from the ANRS-PEDIACAM cohort in Cameroon
Source: PLoS One. 2019 Jul 18;14(7):e0219960. doi: 10.1371/journal.pone.0219960 (PMC6638950; doi:10.1371/journal.pone.0219960)
Supplement: S1 Table — (DOCX) [file pone.0219960.s001.docx]

# S1 Table. Multivariable model, GEE identity with exchangeable correlation structure, describing the factors associated with the evolution of WAZ in the ANRS-PEDIACAM cohort (with and without taking into account biases due to missing data on the WAZ), Cameroun, Nov. 2007- Dec.2015.

|  | **Multivariable analysis without IMR**  **N=5091 visits by 607 children** | | | | |  | **Multivariable analysis with IMR: SE, L, U et P obtained by bootstrap*** | | | | |  |
| --- | --- | --- | --- | --- | --- | --- | --- | --- | --- | --- | --- | --- |
|  | **Coef** | **SE** | **L** | **U** | **P** |  | **Coef** | **SE** | **L** | **U** | **P** |  |
| IMR |  |  |  |  |  |  | -0.39 | 0.169 | -0.74 | -0.07 | 0.022 |  |
| Clinical site (ref= MCH/MCC-CBF) | |  |  |  |  |  |  |  |  |  |  |  |
| LH | -0.03 | 0.102 | -0.23 | 0.17 | 0.770 |  | 0.00 | 0.084 | -0.16 | 0.16 | 0.962 |  |
| EHC | -0.08 | 0.084 | -0.24 | 0.09 | 0.351 |  | -0.11 | 0.066 | -0.24 | 0.01 | 0.080 |  |
| Sex: Female | 0.02 | 0.075 | -0.13 | 0.17 | 0.793 |  | 0.04 | 0.062 | -0.07 | 0.16 | 0.478 |  |
| Age | 0.17 | 0.037 | 0.10 | 0.24 | <.001 |  | 0.27 | 0.060 | 0.16 | 0.38 | <.001 |  |
| Age^2^ | -0.02 | 0.007 | -0.03 | 0.00 | 0.014 |  | -0.03 | 0.008 | -0.04 | -0.01 | <.001 |  |
| Multiple birth | -0.69 | 0.220 | -1.12 | -0.26 | 0.002 |  | -0.70 | 0.172 | -1.02 | -0.36 | <.001 |  |
| Children’s groupe (ref= HEU) |  |  |  |  |  |  |  |  |  |  |  |  |
| HI | -0.32 | 0.137 | -0.59 | -0.06 | 0.018 |  | -0.42 | 0.122 | -0.66 | -0.18 | <.001 |  |
| HIL | -0.52 | 0.128 | -0.77 | -0.27 | <.001 |  | -0.66 | 0.120 | -0.90 | -0.43 | <.001 |  |
| HUU | 0.33 | 0.096 | 0.14 | 0.52 | 0.001 |  | 0.27 | 0.080 | 0.10 | 0.42 | <.001 |  |
| Chronic pathologies | -0.48 | 0.117 | -0.71 | -0.25 | <.001 |  | -0.48 | 0.089 | -0.64 | -0.30 | <.001 |  |
| Anemia | -0.12 | 0.035 | -0.19 | -0.06 | <.001 |  | -0.12 | 0.027 | -0.17 | -0.06 | <.001 |  |
| CD4 <_25% | -0.12 | 0.046 | -0.21 | -0.03 | 0.008 |  | -0.09 | 0.037 | -0.17 | -0.02 | 0.028 |  |
| Professional activity of the mother (ref=paid activity) | | | | |  |  |  |  |  |  |  | |
| Training/student | -0.19 | 0.109 | -0.40 | 0.03 | 0.087 |  | -0.16 | 0.082 | -0.33 | 0.00 | 0.052 |  |
| Housewife/unemployed | -0.21 | 0.091 | -0.39 | -0.04 | 0.019 |  | -0.21 | 0.070 | -0.36 | -0.08 | <.001 |  |
| Developmental delay | -0.67 | 0.086 | -0.84 | -0.50 | <.001 |  | -0.66 | 0.069 | -0.80 | -0.53 | <.001 |  |
| **General signs during the visit** | **-0.09** | **0.058** | **-0.20** | **0.03** | **0.130** |  | **-0.09** | **0.041** | **-0.17** | **-0.01** | **0.032** |  |
| **Breastfeeding** | **0.06** | **0.050** | **-0.03** | **0.16** | **0.201** |  | **0.14** | **0.057** | **0.04** | **0.26** | **0.008** |  |
| Diarrhea | -0.25 | 0.065 | -0.37 | -0.12 | <.001 |  | -0.23 | 0.049 | -0.32 | -0.13 | <.001 |  |
| SGAG | -0.77 | 0.171 | -1.11 | -0.44 | <.001 |  | -0.80 | 0.114 | -1.02 | -0.59 | <.001 |  |
| **Mother’s level of education (ref=higher)** | | | |  |  |  |  |  |  |  |  |  |
| **Secondary** | **-0.23** | **0.100** | **-0.43** | **-0.04** | **0.020** |  | **-0.23** | **0.077** | **-0.37** | **-0.08** | **<.001** |  |
| **Primary** | **-0.52** | **0.144** | **-0.80** | **-0.24** | **<.001** |  | **-0.52** | **0.117** | **-0.74** | **-0.31** | **<.001** |  |
| Electricity supply at home | 0.64 | 0.208 | 0.23 | 1.04 | 0.002 |  | 0.63 | 0.172 | 0.32 | 0.98 | <.001 |  |
| **Water supply at home** | **0.13** | **0.084** | **-0.04** | **0.29** | **0.129** |  | **0.16** | **0.073** | **0.02** | **0.30** | **0.028** |  |

^*^standard errors (SE) and confidence intervalle (L,U) obtained based on 500 replications bootstrap, due to the introduction of the IMR in the model; IMR: Inverse Mills ratio obtained from the residuals of the first stage model; Coef: coefficients; L: Lower bound of the confidence interval; U: Upper bound of the confidence interval; P: Pvalue; HI: HIV infected followed since birth; HIL: HIV infected diagnosed before 7 months old; HEU: HIV uninfected born to infected mothers; HUU: HIV uninfected born to uninfected mothers; MCH/MCC-CBF: Maternity of the Central hospital/Mother and Child Center of the Chantal Biya Foundation; LH: Laquintinie Hospital; EHC: Essos Hospital Center ; SGAG: small-for-gestational age and gender; WAZ: Weight-for-age Zscore.
