# Supplementary material for: Early treated HIV-infected children remain at risk of growth retardation during the first five years of life: Results from the ANRS-PEDIACAM cohort in Cameroon
Source: PLoS One. 2019 Jul 18;14(7):e0219960. doi: 10.1371/journal.pone.0219960 (PMC6638950; doi:10.1371/journal.pone.0219960)
Supplement: S2 Table — (DOCX) [file pone.0219960.s002.docx]

# S2 Table. Multivariable model, GEE identity with exchangeable correlation structure, describing the factors associated with the evolution of LAZ in the ANRS-PEDIACAM cohort (with and without taking into account biases due to missing data on the LAZ), Cameroun, Nov. 2007- Dec.2015.

|  | Multivariable analysis without IMR  N=4966 visits by 605 children | | | | |  | Multivariable analysis with IMR: SE, L, U et P obtained by bootstrap* | | | | |
| --- | --- | --- | --- | --- | --- | --- | --- | --- | --- | --- | --- |
|  | **Coef** | **SE** | **L** | **U** | **P** |  | **Coef** | **SE** | **L** | **U** | **P** |
| IMR |  |  |  |  |  |  | 0.14 | 0.142 | -0.13 | 0.43 | 0.314 |
| Clinical site (ref= MCH/MCC-CBF) | |  |  |  |  |  |  |  |  |  |  |
| LH | -1.01 | 0.115 | -1.24 | -0.78 | <.001 |  | -1.02 | 0.092 | -1.20 | -0.84 | <.001 |
| EHC | -0.14 | 0.097 | -0.33 | 0.06 | 0.164 |  | -0.12 | 0.077 | -0.26 | 0.03 | 0.120 |
| Sex: Female | 0.34 | 0.085 | 0.17 | 0.50 | <.001 |  | 0.33 | 0.068 | 0.19 | 0.46 | <.001 |
| Age | -0.70 | 0.055 | -0.80 | -0.59 | <.001 |  | -0.72 | 0.049 | -0.82 | -0.62 | <.001 |
| Age^2^ | 0.17 | 0.010 | 0.15 | 0.19 | <.001 |  | 0.17 | 0.009 | 0.15 | 0.18 | <.001 |
| Children’s groupe (ref= HEU) |  |  |  |  |  |  |  |  |  |  |  |
| HI | -0.59 | 0.169 | -0.93 | -0.26 | <.001 |  | -0.56 | 0.133 | -0.81 | -0.29 | <.001 |
| HIL | -0.59 | 0.159 | -0.91 | -0.28 | <.001 |  | -0.56 | 0.128 | -0.83 | -0.34 | <.001 |
| HUU | 0.20 | 0.103 | -0.01 | 0.40 | 0.057 |  | 0.20 | 0.078 | 0.05 | 0.36 | 0.020 |
| Anemia | -0.27 | 0.090 | -0.44 | -0.09 | 0.003 |  | -0.28 | 0.067 | -0.41 | -0.15 | <.001 |
| HI: Anemia | 0.30 | 0.167 | -0.02 | 0.63 | 0.068 |  | 0.31 | 0.132 | 0.05 | 0.58 | 0.018 |
| HIL: Anemia | 0.26 | 0.128 | 0.01 | 0.51 | 0.041 |  | 0.26 | 0.096 | 0.07 | 0.47 | 0.002 |
| HUU: Anemia | 0.18 | 0.122 | -0.06 | 0.42 | 0.147 |  | 0.18 | 0.089 | 0.01 | 0.37 | 0.038 |
| Multiple birth | -0.64 | 0.247 | -1.12 | -0.15 | 0.010 |  | -0.63 | 0.207 | -1.02 | -0.21 | 0.006 |
| Chronic pathologie | -0.31 | 0.131 | -0.56 | -0.05 | 0.020 |  | -0.31 | 0.099 | -0.51 | -0.14 | 0.004 |
| Developmental delay Inclusion | -1.01 | 0.228 | -1.46 | -0.56 | <.001 |  | -1.02 | 0.154 | -1.32 | -0.69 | <.001 |
| Small size at birth | -0.50 | 0.230 | -0.96 | -0.03 | 0.036 |  | -0.50 | 0.092 | -0.67 | -0.30 | <.001 |
| Diarrhae | 0.01 | 0.084 | -0.16 | 0.17 | 0.945 |  | -0.02 | 0.072 | -0.16 | 0.12 | 0.724 |
| Home change | -0.28 | 0.063 | -0.40 | -0.15 | <.001 |  | -0.28 | 0.041 | -0.35 | -0.19 | <.001 |
| Professional activity of the mother (ref=paid activity) | | | |  |  |  |  |  |  |  |  |
| Training/student | 0.01 | 0.117 | -0.21 | 0.24 | 0.902 |  | 0.00 | 0.095 | -0.18 | 0.20 | 0.976 |
| Housewife/unemployed | -0.30 | 0.101 | -0.50 | -0.10 | 0.003 |  | -0.30 | 0.076 | -0.44 | -0.15 | <.001 |
| Mother’s level of education (ref=higher) | | | |  |  |  |  |  |  |  |  |
| Secondary | -0.20 | 0.112 | -0.42 | 0.02 | 0.076 |  | -0.21 | 0.087 | -0.38 | -0.03 | 0.010 |
| Primary | -0.39 | 0.169 | -0.72 | -0.06 | 0.021 |  | -0.40 | 0.132 | -0.67 | -0.14 | 0.002 |
| Electricity supply at home | 0.64 | 0.206 | 0.24 | 1.04 | 0.002 |  | 0.64 | 0.163 | 0.35 | 0.98 | 0.002 |
| Water supply at home | 0.26 | 0.096 | 0.07 | 0.45 | 0.007 |  | 0.25 | 0.076 | 0.10 | 0.39 | <.001 |

^*^standard errors (SE) and confidence intervalle (L,U) obtained based on 500 replications bootstrap, due to the introduction of the IMR in the model; IMR: Inverse Mills ratio obtained from the residuals of the first stage model; Coef: coefficients; L: Lower bound of the confidence interval; U: Upper bound of the confidence interval; P: Pvalue; HI: HIV infected followed since birth; HIL: HIV infected diagnosed before 7 months old; HEU: HIV uninfected born to infected mothers; HUU: HIV uninfected born to uninfected mothers; MCH/MCC-CBF: Maternity of the Central hospital/Mother and Child Center of the Chantal Biya Foundation; LH: Laquintinie Hospital; EHC: Essos Hospital Center ; SGAG: small-for-gestational age and gender; LAZ: Length-for-age Zscore.
